# Serum triglycerides level is independently associated with renal outcomes in patients with non-dialysis chronic kidney disease: Results from KNOW-CKD study

**DOI:** 10.3389/fnut.2022.1037618

**Published:** 2022-11-24

**Authors:** Sang Heon Suh, Tae Ryom Oh, Hong Sang Choi, Chang Seong Kim, Eun Hui Bae, Kook-Hwan Oh, Seung Hyeok Han, Seong Kwon Ma, Soo Wan Kim

**Affiliations:** ^1^Department of Internal Medicine, Chonnam National University Medical School and Chonnam National University Hospital, Gwangju, South Korea; ^2^Department of Internal Medicine, Seoul National University Hospital, Seoul, South Korea; ^3^Department of Internal Medicine, College of Medicine, Institute of Kidney Disease Research, Yonsei University, Seoul, South Korea

**Keywords:** chronic kidney disease, dyslipidemia, end-stage renal disease, estimated glomerular filtration rate, triglycerides

## Abstract

To investigate whether high serum triglycerides (TG) level is associated with adverse renal outcomes in patients with non-dialysis chronic kidney disease (CKD), a total of 2,158 subjects from a prospective cohort study (Korean Cohort Study for Outcome in Patients With Chronic Kidney Disease) were divided into the quartile by serum TG level. The primary outcomes were composite renal events, which is defined as a composite of decline of kidney function (the first occurrence of > 50% decline of estimated glomerular filtration rate or doubling of serum creatinine from the baseline) or onset of end-stage renal disease (initiation of dialysis or kidney transplantation). During the median follow-up of 6.940 years, the cumulative incidence of composite renal event was significantly differed by serum TG level in Kaplan–Meier curve analysis (P < 0.001, by Log-rank test). Cox regression analysis demonstrated that, compared to that of the 1st quartile, the risk of composite renal event was significantly higher in the 4th quartile (adjusted hazard ratio 1.433, 95% confidence interval 1.046 to 1.964). The association between high serum TG level and adverse renal outcome remained consistent in the cause-specific hazard model. Subgroup analyses revealed that the association is modified by age, estimated glomerular filtration rate, and spot urine albumin-to-creatinine ratio. In conclusion, high serum TG level is independently associated with adverse renal outcomes in patients with non-dialysis CKD. Interventional studies are warranted to determine whether lowering serum TG levels may alter the natural course of CKD.

## Introduction

Dyslipidemia is commonly accompanied by chronic kidney disease (CKD) (1–3), and is a potentially modifiable cardiovascular (CV) risk factor (4). The clinical practice guideline for lipid management in CKD recommends a pharmacological approach with use of statins to mainly lower low-density lipoprotein cholesterol (LDL-C) level (4). Yet, dyslipidemia in CKD is characterized with high serum triglycerides (TG) and low high-density lipoprotein cholesterol (HDL-C) level (1–3). Moreover, high TG or low HDL-C level, or high TG-to-HDL-C ratio has been associated with increased risk of cardiovascular (CV) events and all-cause mortality in patients with CKD (5, 6) as well as in general population (7–9).

Mounting evidence now suggest that dyslipidemia may also contribute to the development and progression of CKD (10–14). Atherosclerosis Risk in Communities study that assessed 12,728 participants reported that high TGs and low HDL-C, but not LDL-C, levels increased the risk of renal dysfunction (15). A prospective cohort study that analyzed 4,483 initially healthy men from the Physicians' Health Study demonstrated that various indices in lipid metabolism, including elevated total cholesterol, high non-HDL-C, high ratio of total cholesterol to HDL-C, and low HDL-C, significantly increased the risk of renal dysfunction (16). A community-based, longitudinal cohort study of 2,585 participants from Framingham Offspring Study showed that HDL-C level is inversely associated with the risk of new-onset CKD among those free of CKD at the baseline (17). Importantly, a recent study conducted in Japan proved that higher TG-to-HDL-C ratio was associated with more rapid decline of estimated glomerular filtration rate (eGFR) in participants with CKD (1).

Indeed, it seems obvious that dyslipidemia is associated with increased risk of incident CKD among the general population, and that a certain abnormal lipid index, such as TG-to-HDL-C ratio, is also associated with rapid progression of CKD. Yet, few studies so far evaluated an independent association of serum TG level with adverse renal outcomes in patients with non-dialysis CKD (3, 18). Therefore, we here aimed to investigate the association between serum TG level and renal outcomes in patients with non-dialysis CKD. We performed a series of sensitivity analyses to validate our findings. Finally, we conducted a series of subgroup analyses to examine whether the association between serum TG level and renal outcomes might be modified clinical contexts.

## Methods

### Study design

**Figure 1:**
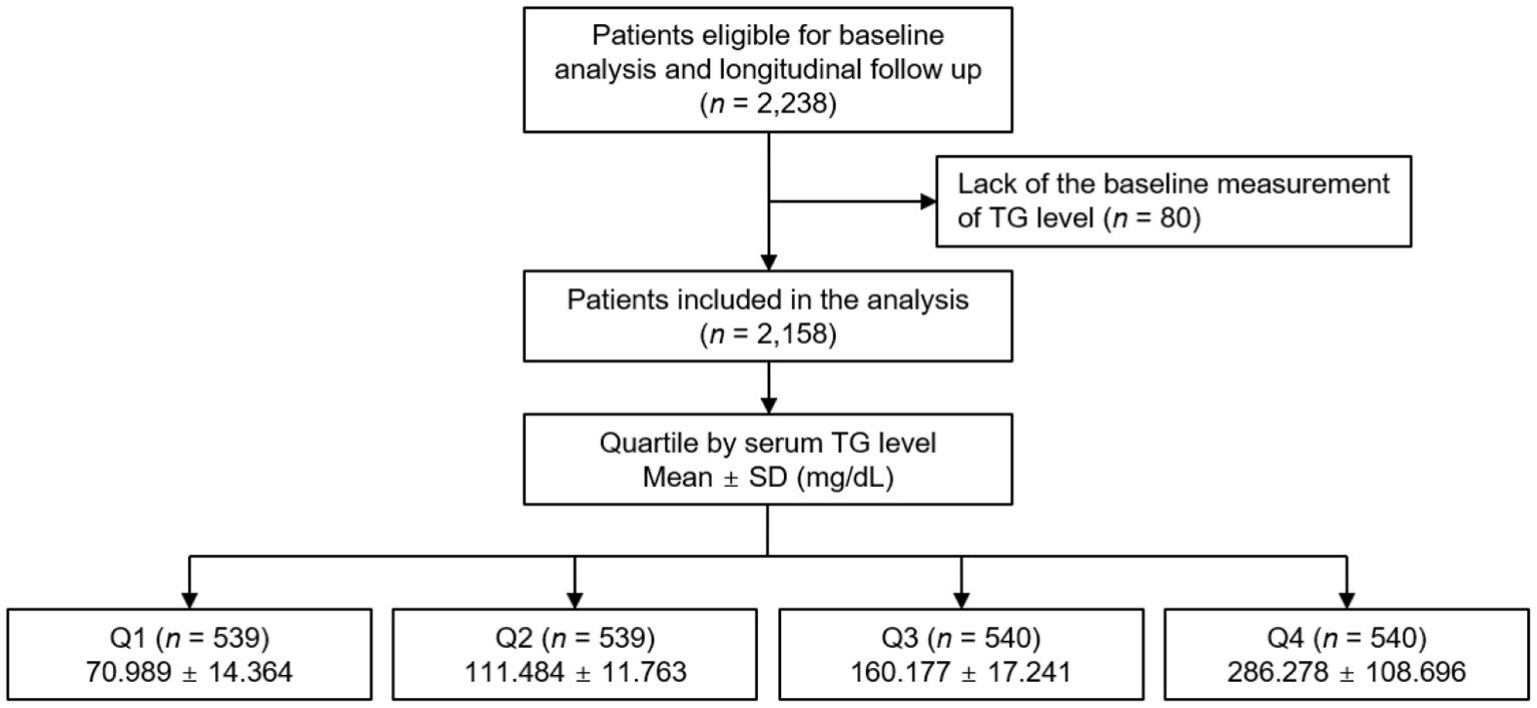
Flow diagram of the study participants. SD, standard deviation; TG, triglyceride; Q1, 1st quartile; Q2, 2nd quartile; Q3, 3rd quartile; Q4, 4th quartile.

The Korean Cohort Study for Outcomes in Patients With Chronic Kidney Disease (KNOW-CKD) is a nationwide prospective cohort study involving nine tertiary-care general hospitals in Korea (NCT01630486 at http://www.clinicaltrials.gov) (19). Korean patients, aged between 20 and 75 years, with CKD from stage 1 to pre-dialysis stage 5, who voluntarily provided informed consent were enrolled from 2011 through 2016. The study was conducted in accordance with the principles of the Declaration of Helsinki. The study protocol was approved by the institutional review boards of participating centers, including at Seoul National University Hospital, Yonsei University Severance Hospital, Kangbuk Samsung Medical Center, Seoul St.Mary's Hospital, Gil Hospital, Eulji General Hospital, Chonnam National University Hospital, and Busan Paik Hospital. All participants had been under close observation, and participants who experienced study out-comes were reported by each participating center. Among 2,238 who were longitudinally followed up, excluding those lacking the baseline measurement of serum TG level, a total of 2,158 subjects were finally included for the analyses (Figure 1). The study observation period ended on March 31, 2021. The median follow-up duration was 6.940 years.

### Data collection from participants

Demographic information was collected from all eligible participants, as previously described (20). The methods for anthropometric measures, such as height, weight, waist circumference (WC), body mass index (BMI), and systolic and diastolic blood pressures (SBP and DBP), are also previously described (21). Venous samples were collected following overnight fasting, to determine hemoglobin, creatinine (Cr), TG, HDL-C, LDL-C, total cholesterol, fasting glucose, high-sensitivity C-reactive protein (hs-CRP), albumin, and 25-hydroxyvitamin D [25(OH) vitamin D] levels at the baseline. Chronic Kidney Disease Epidemiology Collaboration equation was adopted to calculate eGFR (22). CKD stages were determined by the Kidney Disease Improving Global Outcomes guidelines (23). Spot urine albumin-to-Cr ratio (ACR) was measured in random, preferably second-voided, urine samples. The coronary artery calcium score (CACS) score was determined using Agatston unit (AU) on a digital radiologic workstation at the baseline (24), following electrocardiography-gated coronary multi-detector computed tomography scans, based on the standard protocol of each center.

### Exposure and study outcome

The exposure of primary interest was serum TG level, which was used as a categorical variable. The subjects were divided into the quartile (Q1 [20–92 mg/dL], Q2 [92–132 mg/dL], Q3 [133–193 mg/dL] and Q4 [194–909 mg/dL]) by serum TG level (Figure 1). The primary outcomes of interest were composite renal events. Composite renal events included decline of kidney function (the first occurrence of > 50% decline of eGFR or doubling of serum Cr from the baseline) and onset of end-stage renal disease (ESRD, initiation of dialysis or kidney transplantation) during follow-up periods. The secondary outcomes were the components of composite renal events (decline of kidney function and onset of ESRD), analyzed separately. In cases that decline of kidney function event and onset of ESRD event simultaneously occurred in a visit, both events were counted.

### Statistical analysis

Continuous variables were expressed as mean ± standard deviation or median [interquartile range]. Categorical variables were expressed as number of participants and percentage. Kolmogorov-Smirnov test was conducted to validate normality of distribution. To compare the baseline characteristics by serum TG level, one-way analysis of variance and χ2 test were used for continuous and categorical variates, respectively. eGFR was calculated by measuring serum Cr level at 0, 6, and 12 months and then yearly thereafter up to 9 years. eGFR decline rate per year were calculated using a generalized linear mixed model, where, excluding the subjects with serum Cr measurement less than three times during the follow-up period, a total of 1,890 (87.5%) patients were included. Cumulative incidences of outcome events were estimated using Kaplan–Meier analyses, and were compared using log-rank test. The participants with any missing data were excluded for further analyses. To address the association between serum TG level and study outcomes, we used Cox proportional hazard regression models. Patients with follow-up loss were censored at the time of the last visit. Models were constructed after adjusting for the following variables. Model 1 represents crude hazard ratios (HRs). Model 2 was adjusted for age, sex, BMI, WC, medication [diuretics, statins angiotensin-converting enzyme inhibitor/angiotensin receptor blockers (ACEi/ARBs), number of antihypertensive drugs], SBP and DBP, primary renal disease, smoking history, and Charlson comorbidity index. Model 3 was further adjusted for CACS, hemoglobin, 25(OH) vitamin D, albumin, fasting glucose, LDL-C, HDL-C, total cholesterol, and hs-CRP. Model 4 was additionally adjusted for CKD stage and spot urine ACR. The results of Cox proportional hazard models were presented as HRs and 95% confidence intervals (CIs). Restricted cubic splines were used to visualize the association between serum TG level as a continuous variable and HRs for study outcomes. To validate our findings, we performed sensitivity analyses. For this purpose, first, we exclude the subjects with eGFR ≥ 90 mL/min./1.73 m^2^ (CKD stage 1), because the subjects with eGFR ≥ 90 mL/min./1.73 m^2^ are considered close to normal kidney function, and may not represent CKD population well. Second, for another sensitivity analysis, we excluded the subjects with eGFR <15 mL/min./1.73 m^2^ (CKD stage 5), because the subjects with eGFR <15 mL/min./1.73 m^2^ are relatively small in number, and may exaggerate the association between serum TG level and study outcomes due to far advanced CKD. Third, the association between TG-to-HDL-C ratio and the primary study outcomes was evaluated, to examine whether the association between a previously proven lipid index and the study outcome is reproducible in the participants of the current study. Fourth, we assessed cause-specific HRs for the primary and secondary study outcomes by serum TG levels, where death before reaching the composite renal event was considered a competing risk and treated as censoring. We also estimated the cumulative outcome curves by cumulative incidence function with Gray's test. To examine whether the association of serum TG level with study outcomes is modified by certain clinical contexts, we conducted pre-specified subgroup analyses. Subgroups were defined by age [ <60 *versus* (*vs*.) ≥ 60 years], sex (male *vs*. female), BMI (<23 *vs*. ≥ 23 kg/m^2^), eGFR (<45 *vs*. ≥ 45 mL/min/1.73 m^2^)), and spot urine ACR (<300 *vs*. ≥ 300 mg/g). The cut-off value for eGFR was determined because the number of subjects with eGFR ≥ 45 ml/min/1.73 m^2^) (*n* = 1,113) and with eGFR <45 ml/min/1.73 m^2^) (*n* = 1,045) was almost equal. The cut-off value for spot urine ACR was determined according to the definition of macroalbuminuria. Two-sided *P* < 0.05 were considered statistically significant. Statistical analysis was performed using SPSS for Windows version 22.0 (IBM Corp., Armonk, NY) and R (version 4.1.1; R project for Statis-tical Computing, Vienna, Austria).

## Results

### Baseline characteristics

To describe the baseline characteristics, the study participants were divided into the quartile by serum TG level (Table 1). The mean age of the participants was lower in the 1st quartile (Q1) than in the 2nd (Q2), 3rd (Q3) and 4th (Q4) quartile. The proportion of male participants significantly increased as serum TG level increased. The subjects in Q1 tend to have low Charlson comorbidity index, compared to those in Q4. The history of diabetes mellitus was least and most frequent in Q1 (19.6%) and Q4 (31.5%), respectively, whereas the prevalence of polycystic kidney disease was lowest and highest in Q4 (8.2%) and Q1 (23.6%), respectively. The proportion of non-smoker was highest in Q1. The proportions of the subjection on medication of ACEi/ARBs, diuretics, no less than three antihypertensive drugs or statins were lowest in Q1, and tended to increase as serum TG level increased. BMI, WC, SBP, and DBP were also lowest in Q1, and increased as serum TG level in-creased. The proportion of the subjection without coronary artery calcification (CACS = 0) was highest in Q1. Hemoglobin, total cholesterol, LDL-C, fasting glucose, hs-CRP, and spot urine ACR were highest in Q4, whereas HDL-C and 25(OH) vitamin D were highest in Q1. Most importantly, eGFR at the baseline significantly differed by serum TG level, as eGFR was best and worst preserved in Q1 and Q4, respectively. Accordingly, the proportion of the subjects with advanced CKD stages were relatively higher in Q4. In the overall, the analysis of the baseline characteristics unveiled that high serum TG level is associated with unfavorable clinical features.

**Table 1:** Baseline characteristics of study participants by serum TG levels.

	**Serum TG level**				
	**Q1**	**Q2**	**Q3**	**Q4**	***P* value**
Follow-up duration (year)	6.484 ± 2.537	6.243 ± 2.525	6.189 ± 2.607	6.245 ± 2.431	0.232
Age (year)	52.021 ± 13.317	54.458 ± 12.048	54.619 ± 11.978	53.826 ± 11.600	0.004
Male	294 (55.1)	309 (57.8)	349 (65.1)	368 (68.3)	<0.001
Charlson comorbidity index					0.001
0 – 3	412 (77.2)	400 (74.8)	359 (67.0)	356 (66.0)	
4 – 5	113 (21.2)	127 (23.7)	168 (31.3)	174 (32.3)	
6 – 7	9 (1.7)	8 (1.5)	8 (1.5)	9 (1.7)	
≥ 8	0 (0.0)	0 (0.0)	1 (0.2)	0 (0.0)	
Primary renal disease					<0.001
DM	106 (19.6)	117 (21.9)	153 (28.6)	170 (31.5)	
HTN	82 (15.4)	106 (19.8)	117 (21.9)	118 (21.9)	
GN	177 (33.1)	177 (33.1)	158 (29.5)	169 (31.4)	
TID	4 (0.7)	5 (0.9)	2 (0.4)	3 (0.6)	
PKD	126 (23.6)	96 (17.9)	72 (13.5)	44 (8.2)	
Others	39 (7.3)	34 (6.4)	33 (6.2)	35 (6.5)	
Smoking status					<0.001
Non-smoker	310 (58.1)	310 (57.9)	268 (50.2)	258 (47.9)	
Ex-smoker	53 (9.9)	76 (14.2)	96 (18.0)	113 (21.0)	
Current smoker	171 (32.0)	149 (27.9)	170 (31.8)	168 (31.2)	
Medication					
ACEi/ARBs	432 (80.9)	458 (85.6)	473 (88.2)	476 (88.3)	0.001
Diuretics	117 (21.9)	151 (28.2)	183 (34.1)	230 (42.7)	<0.001
Number of anti-HTN drugs ≥ 3	116 (21.7)	132 (24.7)	180 (33.6)	199 (36.9)	<0.001
Statins	226 (42.3)	279 (52.1)	303 (56.5)	303 (56.2)	<0.001
BMI (kg/m^2^)	23.347 ± 3.156	24.101 ± 3.084	25.121 ± 3.400	25.891 ± 3.430	<0.001
WC (cm)	83.523 ± 9.454	85.782 ± 8.797	89.470 ± 9.420	91.505 ± 9.099	<0.001
SBP (mmHg)	124.798 ± 15.655	127.419 ± 16.382	128.781 ± 14.628	130.578 ± 17.724	<0.001
DBP (mmHg)	75.552 ± 11.387	76.809 ± 11.030	76.817 ± 10.346	78.539 ± 11.534	<0.001
CACS (AU)					<0.001
0	289 (56.9)	263 (52.2)	217 (43.5)	200 (40.0)	
> 0, ≤ 400	168 (33.1)	192 (38.1)	220 (44.1)	234 (46.8)	
> 400, ≤ 1,000	30 (5.9)	32 (6.3)	35 (7.0)	36 (7.2)	
> 1,000	21 (4.1)	17 (3.4)	27 (5.4)	30 (6.0)	
Laboratory findings					
Hemoglobin (g/dL)	12.562 ± 1.847	12.771 ± 2.028	12.835 ± 2.052	13.205 ± 2.114	<0.001
Albumin (g/dL)	4.198 ± 0.358	4.203 ± 0.382	4.153 ± 0.445	4.150 ± 0.509	0.066
Total cholesterol (mg/dL)	163.101 ± 34.909	171.131 ± 36.428	173.426 ± 37.202	189.618 ± 43.611	<0.001
LDL-C (mg/dL)	90.228 ± 27.097	98.237 ± 30.449	99.627 ± 32.967	99.818 ± 35.608	<0.001
HDL-C (mg/dL)	57.680 ± 16.291	51.295 ± 14.383	46.019 ± 12.885	42.237 ± 13.497	<0.001
Fasting glucose (mg/dL)	100.066 ± 23.889	106.409 ± 34.417	111.017 ± 33.990	127.366 ± 55.756	<0.001
25(OH) Vitamin D (ng/mL)	19.521 ± 9.159	17.731 ± 7.151	17.918 ± 7.789	15.964 ± 7.026	<0.001
hs-CRP (mg/dL)	0.500 [0.200, 1.350]	0.500 [0.200, 1.500]	0.600 [0.250, 1.700]	0.980 [0.400, 2.00]	0.913
Spot urine ACR (mg/g)	203.959 [38.478, 643.516]	300.545 [54.264, 818.582]	405.691 [59.429, 1,207.620]	526.679 [150.419, 1,647.123]	<0.001
eGFR (mL/min./1.73 m^2^)	55.682 ± 32.118	50.401 ± 30.247	48.238 ± 28.704	48.895 ± 28.032	<0.001
CKD stages					<0.001
Stage 1	119 (22.3)	86 (16.1)	70 (13.1)	67 (12.4)	
Stage 2	113 (21.2)	92 (17.2)	114 (21.3)	88 (16.3)	
Stage 3a	86 (16.1)	101 (18.9)	72 (13.4)	98 (18.2)	
Stage 3b	90 (16.9)	114 (21.3)	121 (22.6)	128 (23.7)	
Stage 4	94 (17.6)	106 (19.8)	118 (22.0)	132 (24.5)	
Stage 5	32 (6.0)	36 (6.7)	41 (7.6)	26 (4.8)	

Values for categorical variables are given as number (percentage); values for continuous variables, as mean ± standard deviation or median [in-terquartile range].

ACEi, angiotensin converting enzyme inhibitor; ACR, albumin-to-creatinine ratio; ARB, angiotensin receptor blocker; AU, Agatston unit; BMI, body mass index; CKD, chronic kidney disease; CACS, coronary artery calcium score; Cr, creatinine; DBP, diastolic blood pressure; DM, diabetes mellitus; eGFR, estimated glomerular filtration rate; GN, glomerulonephritis; HDL-C, high density lipoprotein cholesterol; hs-CRP, high-sensitivity C-reactive protein; HTN, hypertension; LDC-C, low density lipoprotein cholesterol; PKD, polycystic kidney disease; SBP, systolic blood pressure; TG, triglycerides; TID, tubulointerstitial disease; WC, waist circumference.

### Association of serum TG level with renal outcomes in patients with non-dialysis CKD

**Figure 2:**
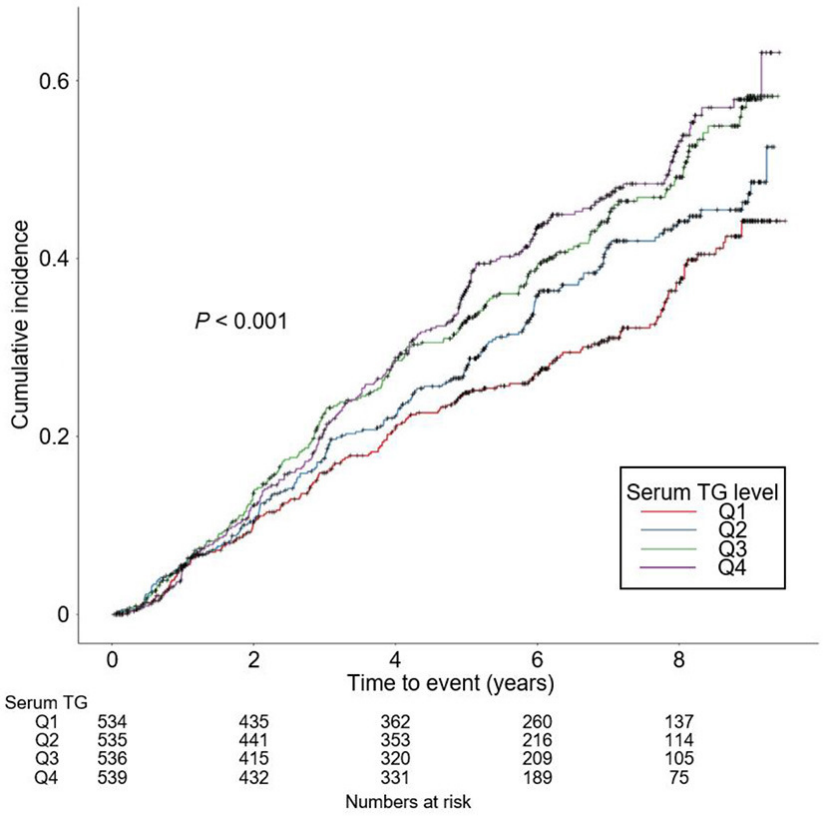
Kaplan-Meier analysis for cumulative incidence of composite renal event by serum TG levels. *P* value by Log-rank test. TG, triglycerides; Q1, 1st quintile; Q2, 2nd quintile; Q3, 3rd quintile; Q4, 4th quintile.

**Figure 3:**
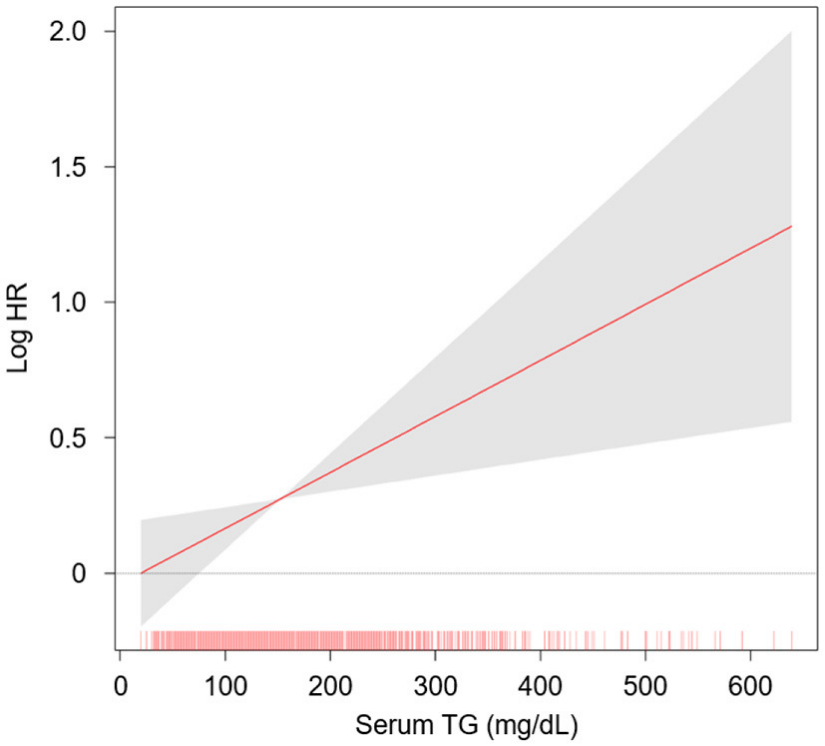
Restricted cubic spline of serum TG level on composite renal event. Adjusted HR of serum TG level as a continuous variable for composite renal events is depicted. The model was adjusted for age, sex, BMI, WC, medication (diuretics, statins, ACEi/ARBs, number of antihypertensive drugs), SBP and DBP, primary renal disease, smoking history, Charlson comorbidity index, CACS, hemoglobin, 25(OH) vitamin D, albumin, fasting glucose, LDL-C, HDL-C, total cholesterol, hs-CRP, CKD stage and spot urine ACR. HR, hazard ratio; TG, triglycerides.

The rates of renal function decline per year by serum TG levels significantly differed by serum TG levels (Supplementary Figure S1), as eGFR decline rate of Q4 was significantly more rapid than that of Q1 (*P* < 0.05, by one-way ANOVA with Scheffe's *post-hoc* analyses). To compare the cumulative incidences of composite renal event (Figure 2), decline of kidney function (Supplementary Figure S2) and onset of ESRD (Supplementary Figure S3), Kaplan–Meier curve analyses were conducted. The risks of composite renal event, decline of kidney function and onset of ESRD were significantly differed by serum TG level (all *P* < 0.001, by Log-rank test). To define the independent association of serum TG level with study outcomes, Cox regression models were analyzed. The risk of composite renal event was significantly higher in Q4 (adjusted HR 1.433, 95% CI 1.046 to 1.964), compared to that of Q1 (Table 2). The risk of decline of kidney function (adjusted HR 1.699, 95% CI 1.167 to 2.474), but not that of onset of ESRD (adjusted HR 1.272, 95% CI 0.891 to 1.815), was significantly higher in Q4, compared to that of Q1 (Supplementary Table S1). Restricted cubic splines visualized stringent linear correlations of serum TG level with the risks of composite renal event (Figure 3), decline of kidney function (Supplementary Figure S4) and onset of ESRD (Supplementary Figure S5).

**Table 2:** Cox regression analysis of serum TG levels for primary outcome.

	**Serum TG level**	**Events, n (%)**	**Model 1**		**Model 2**		**Model 3**		**Model 4**	
			**HR (95%Cis)**	***P* value**	**HR (95%Cis)**	***P* value**	**HR (95%Cis)**	***P* value**	**HR (95%Cis)**	***P* value**
Composite renal event	Q1	167 (31.3)	Reference		Reference		Reference		Reference	
	Q2	193 (36.1)	1.222 (0.993, 1.503)	0.058	1.273 (1.024, 1.582)	0.030	1.388 (1.093, 1.763)	0.007	1.248 (0.978, 1.594)	0.075
	Q3	218 (40.7)	1.479 (1.209, 1.810)	<0.001	1.366 (1.073, 1.665)	0.010	1.475 (1.142, 1.904)	0.003	1.150 (0.888, 1.488)	0.289
	Q4	230 (42.7)	1.578 (1.293, 1.927)	<0.001	1.342 (1.071, 1.681)	0.011	1.759 (1.279, 2.419)	<0.001	1.433 (1.046, 1.964)	0.025

Model 1, unadjusted model. Model 2, model 1 + adjusted for age, sex, BMI, WC, medication (diuretics, ACEi/ARBs, number of antihypertensive drugs), SBP and DBP, primary renal disease, smoking history, and Charlson comorbidity index. Model 3, model 2 + adjusted for CACS, hemoglobin, 25(OH) vitamin D, albumin, fasting glucose, LDL-C, HDL-C, total cholesterol, and hs-CRP. Model 4, model 3 + CKD stage and spot urine ACR. CI, confidence interval; HR, hazard ratio; TG, triglycerides; Q1, 1st quartile; Q2, 2nd quartile; Q3, 3rd quartile; Q4, 4th quartile.

### Sensitivity analyses

To validate the findings, we conducted a series of sensitivity analyses. After excluding the subjects with eGFR ≥ 90 mL/min./1.73 m^2^ (adjusted HR 1.443, 95% CI 1.048 to 1.986), or after excluding the subjects with eGFR <15 mL/min./1.73 m^2^ (adjusted HR 1.517, 95% CI 1.075 to 2.141), the risk of composite renal event remained significantly higher in Q4, compared to that of Q1 (Supplementary Tables S2, S3). The HR for composite renal outcome by se-rum TG-to-HDL-C ratio, which has been previously proven to be associated with renal outcomes, was also significantly higher in Q4 (adjusted HR 1.623, 95% CI 1.106 to 2.383), compared to that of Q1 (Supplementary Table S4). Finally, we analyzed cause-specific hazard model for the primary study outcome by serum TG levels, where the risk of composite renal event was still robustly higher in Q4 (adjusted HR 1.434, 95% CI 1.030 to 1.996), compared to that of Q1 (Table 3). The analyses of cause-specific hazard models for the secondary study outcomes also demonstrated the similar results to that of primary analysis (Supplementary Table S5). The rate of composite renal event by Gray's test for competing risk (*P* < 0.001) was still significantly differed by serum TG levels (Supplementary Figure S6).

**Table 3:** Cause-specific hazard model for primary outcome by serum TG levels.

	**Serum TG level**	**Model 1**		**Model 2**		**Model 3**		**Model 4**	
		**HR (95%CIs)**	***P* value**	**HR (95%CIs)**	***P* value**	**HR (95%CIs)**	***P* value**	**HR (95%CIs)**	***P* value**
Composite renal event	Q1	Reference		Reference		Reference		Reference	
	Q2	1.222 (0.992, 1.505)	0.059	1.272 (1.019, 1.590)	0.033	1.388 (1.079, 1.786)	0.011	1.248 (0.974, 1.600)	0.080
	Q3	1.479 (1.208, 1.811)	<0.001	1.337 (1.064, 1.680)	0.013	1.475 (1.122, 1.938)	0.005	1.150 (0.873, 1.514)	0.319
	Q4	1.579 (1.293, 1.927)	<0.001	1.341 (1.054, 1.708)	0.017	1.759 (1.250, 2.477)	0.001	1.434 (1.030, 1.996)	0.033

Model 1, unadjusted model. Model 2, model 1 + adjusted for age, sex, BMI, WC, medication (diuretics, ACEi/ARBs, number of antihypertensive drugs), SBP and DBP, primary renal disease, smoking history, and Charlson comorbidity index. Model 3, model 2 + adjusted for CACS, hemoglobin, 25(OH) vitamin D, albumin, fasting glucose, LDL-C, HDL-C, total cholesterol, and hs-CRP. Model 4, model 3 + CKD stage and spot urine ACR. Abbreviations: CI, confidence interval; HR, hazard ratio; TG, triglycerides; Q1, 1st quartile; Q2, 2nd quartile; Q3, 3rd quartile; Q4, 4th quartile.

### Subgroup analyses

To address whether the association of serum TG level with study outcomes is modified by certain clinical contexts, we performed pre-specified subgroup analyses. The association between TG level and composite renal event was significantly more prominent in the subjects with age <60 years (*P* for interaction = 0.004), eGFR ≥ 45 mL/min./1.73 m^2^ (*P* for interaction = 0.040), and spot urine ACR ≥ 300 mg/g (*P* for interaction = 0.043) (Table 4). The association between TG level and decline of kidney function was also significantly more prominent in the subjects with age <60 years (*P* for interaction = 0.046), eGFR ≥ 45 mL/min./1.73 m^2^ (*P* for interaction = 0.022), and spot urine ACR ≥ 300 mg/g (*P* for interaction = 0.043) (Supplementary Table S6). The association between TG level and onset of ESRD was not significantly altered by clinical contexts, such as age, sex, BMI, eGFR, or albuminuria (Supplementary Table S7).

**Table 4:** Cox regression analysis of serum TG levels for composite renal events in various subgroups.

	**Serum TG levels**	**Events, n (%)**	**Unadjusted HR (95%CIs)**	***P* for interaction**	**Adjusted HR (95%CIs)**	***P* for interaction**
Age <60 years	Q1	108 (30.3)	Reference	0.067	Reference	0.004
	Q2	120 (34.7)	1.224 (0.944, 1.588)		1.205 (0.880, 1.649)	
	Q3	127 (38.7)	1.492 (1.154, 1.928)		0.988 (0.700, 1.396)	
	Q4	158 (45.9)	1.843 (1.442, 2.355)		1.582 (1.058, 2.366)	
Age ≥ 60 years	Q1	59 (33.3)	Reference		Reference	
	Q2	73 (38.6)	1.195 (0.848, 1.685)		1.597 (1.025, 2.488)	
	Q3	91 (43.8)	1.396 (1.006, 1.938)		1.651 (1.066, 2.556)	
	Q4	147 (75.4)	1.150 (0.814, 1.623)		1.414 (0.789, 2.537)	
Male	Q1	97 (33.0)	Reference	0.142	Reference	0.929
	Q2	108 (35.0)	1.096 (0.833, 1.442)		1.040 (0.743, 1.457)	
	Q3	132 (37.8)	1.298 (0.999, 1.687)		1.011 (0.710, 1.441)	
	Q4	146 (39.7)	1.321 (1.022, 1.709)		1.166 (0.768, 1.769)	
Female	Q1	70 (29.2)	Reference		Reference	
	Q2	85 (37.6)	1.390 (1.013, 1.907)		1.455 (0.997, 2.124)	
	Q3	86 (46.0)	1.758 (1.282, 2.411)		1.343 (0.894, 2.016)	
	Q4	84 (49.1)	2.105 (1.532, 2.893)		1.667 (0.996, 2.790)	
BMI <23 kg/m^2^	Q1	86 (34.5)	Reference	0.884	Reference	0.257
	Q2	77 (39.1)	1.323 (0.972, 1.800)		1.248 (0.836, 1.862)	
	Q3	57 (44.5)	1.700 (1.215, 2.379)		1.169 (0.712, 1.919)	
	Q4	38 (41.3)	1.661 (1.132, 2.437)		1.182 (0.616, 2.268)	
BMI ≥ 23 kg/m^2^	Q1	80 (28.5)	Reference		Reference	
	Q2	116 (34.8)	1.195 (0.899, 1.589)		1.286 (0.917, 1.802)	
	Q3	159 (39.4)	1.449 (1.107, 1.895)		1.334 (0.962, 1.851)	
	Q4	192 (43.2)	1.623 (1.250, 2.107)		1.609 (1.088, 2.379)	
eGFR ≥ 45 mL/min./1.73m^2^	Q1	46 (14.5)	Reference	0.035	Reference	0.040
	Q2	42 (15.1)	1.253 (0.835, 1.882)		1.301 (0.794, 2.130)	
	Q3	33 (12.9)	1.042 (0.668, 1.623)		0.952 (0.537, 1.689)	
	Q4	56 (22.1)	1.876 (1.275, 2.760)		2.170 (1.087, 4.333)	
eGFR <45 mL/min./1.73m^2^	Q1	121 (56.0)	Reference		Reference	
	Q2	146 (57.0)	0.964 (0.757, 1.228)		1.247 (0.931, 1.670)	
	Q3	184 (65.7)	1.200 (0.954, 1.509)		1.205 (0.896, 1.620)	
	Q4	171 (59.8	1.107 (0.877, 1.398)		1.251 (0.871, 1.798)	
Spot urine ACR <300 mg/g	Q1	62 (20.5)	Reference	0.171	Reference	0.043
	Q2	63 (24.9)	1.202 (0.846, 1.706)		1.080 (0.697, 1.672)	
	Q3	50 (22.5)	1.174 (0.808, 1.704)		1.237 (0.741, 2.064)	
	Q4	29 (15.7)	0.857 (0.551, 1.334)		1.157 (0.555, 2.414)	
Spot urine ACR ≥ 300 mg/g	Q1	103 (47.9)	Reference		Reference	
	Q2	126 (48.3)	1.099 (0.847, 1.426)		1.263 (0.925, 1.726)	
	Q3	163 (55.6)	1.292 (1.010, 1.655)		1.003 (0.734, 1.371)	
	Q4	194 (57.7)	1.278 (1.006, 1.623)		1.424 (0.986, 2.056)	

The model was adjusted for age, sex, BMI, WC, medication (diuretics, statins, ACEi/ARBs, number of antihypertensive drugs), SBP and DBP, primary renal disease, smoking history, Charlson comorbidity index, CACS, hemoglobin, 25(OH) vitamin D, albumin, fasting glucose, LDL-C, HDL-C, total cholesterol, hs-CRP, CKD stage and spot urine ACR.

ACR, albumin-to-creatinine ratio; BMI, body mass index; CI, confidence interval; Cr, creatinine; eGFR, estimated glomerular filtration rate; HR, hazard ratio; TG, triglycerides.

## Discussion

In the present study, we found that high serum TG level is independently associated with adverse renal outcomes in patients with non-dialysis CKD. Our finding is robust, because we demonstrated consistent results in a series of sensitivity analyses, including the analysis of cause-specific hazard models, in which the death occurring before reaching the primary outcome was treated as a competing risk and censored. We also observed that the association of serum TG level with renal outcomes is modified by several clinical contexts, as the association was more prominent in the subjects with age <60 years, eGFR ≥ 45 mL/min./1.73 m^2^, and spot urine ACR ≥ 300 mg/g.

Although the precise mechanism for the association between high serum TG level and adverse renal outcomes in patients with CKD is not clearly presented in the current study, some possible explanations might be suggested. TG-rich lipoproteins and accumulation of their oxidation-prone, atherogenic remnants are known to accelerate atherosclerosis (25), triggering atherogenic and pro-inflammatory process in the renal vasculature. In addition, hypertriglyceridemia may promote cellular uptake and accumulation of free fatty acid by the cells in the kidney, such as mesangial cells and macrophages, exerting direct lipotoxicity (26), which is further linked to increased oxidative stress (27, 28), and activation of intrarenal renin-angiotensin system (29, 30). Inversely, it is also possible that serum TG level is elevated in patients with CKD, due to down-regulation of lipoprotein lipase and very low-density lipoprotein receptor in the adipose tissue, skeletal muscle and cardiac muscle (2, 25). Thus, it seems prudent that elevation of serum TG level may be both the cause and consequence of CKD progression.

The current clinical practice guideline for lipid management in CKD does not recommend pharmacologic intervention targeting the reduction of serum TG level, whereas the use of statins is strongly recommended, which effectively alter serum LDL-C level (4). This is primarily attributed to the lack of the firm evidence supporting the efficacy of pharmacological treatment for high serum TG level to reduce CV events, especially in patients with CKD (4). In contrast, the therapeutic efficacy targeting serum TG level to reduce the risk of CKD progression has not been thoroughly reviewed yet. Therefore, interventional studies are warranted to determine whether lowering serum TG levels may alter the natural course of CKD.

A previous paper from the Chronic Renal Insufficiency Cohort (CRIC) Study reported, which analyzed 3,939 adult patients with non-dialysis CKD of mean age 58.2 years and mean eGFR 44.9 mL/min/1.73 m^2^, that serum TG level is not independently associated with progression of CKD (31). A possible reason for the conflicting result from the current study might be the ethnicity, as the CRIC Study was enrolled the participants via the sites in the United States (32), whereas the current study enrolled Koreans resident in South Korea only (19). Moreover, the result was marginal, as adjusted HR was 1.05 with 95% CIs 0.97 to 1.12, which indicates that the trend association would likely have become significant if the number of participants was further increased. Accordingly, a more recent study, which analyzed the association between serum TG level and renal outcomes among 1.6 million veterans in the United States, reported that high serum TG level is associated with rapid decline of eGFR (18). The study, however, included only a small portion of the participants with CKD (~25%), among which more than a half were at CKD stage 3a (18). An observational study from Japan, which included 20,729 individuals with CKD who participated in health checkup, also reported a significant association between elevated serum TG level and CKD progression (3), where the follow-up duration was limited to 2 years, and the magnitude of eGFR decline for the study outcome was modest (> 30% drop in the eGFR from baseline). Collectively, we believe that, compared to the previous studies, the cohort in the present study includes a sufficient number of CKD patients with relatively long follow-up duration, presenting compelling evidence for the association between high serum TG level and the risk of CKD progression.

Our study has several limitations to be acknowledged. First, we cannot determine the casual relation between high serum TG level and CKD progression, because of the observational nature of the current study. However, evidence so far suggests that elevation of serum TG level may be both the cause and consequence of CKD progression (2, 25–30). It should be, therefore, emphasized that further interventional studies are re-quired to determine whether lowering serum TG levels may prevent CKD progression. Second, all the variables were measured once at the baseline. However, the previous observational studies (3, 18), which have the same limitation, reported the findings that are largely in line with ours. We believe that, therefore, the single measurement of the variables does not interfere with the overall results in the present study. Third, as this cohort study enrolled only ethnic Koreans, a precaution is required to extrapolate the data to other populations. Nevertheless, it should be noted that a similar trend has been also reproduced by a study conducted in the United States (18). Fourth, the specific cause of CKD was mainly defined by clinical diagnosis, because the kidney biopsy result was available in only 588 out of 2,158 participants (27.2%). More-over, medication-related kidney disease was not prespecified as a primary renal disease. Fifth, the participants with any missing data were excluded for regression analyses, which may result in the potential bias.

In conclusion, we report that high serum TG level is independently associated with adverse renal outcomes in patients with non-dialysis CKD, and that the association is modified by clinical contexts, such as age, eGFR, and albuminuria. Interventional studies are warranted to determine whether lowering serum TG levels may alter the natural course of CKD.

## Data availability statement

The raw data supporting the conclusions of this article will be made available by the authors, without undue reservation.

## Ethics statement

The study protocol was approved by the Institutional Review Boards of participating centers, including at Seoul National University Hospital, Yonsei University Severance Hospital, Kangbuk Samsung Medical Center, Seoul St. Mary's Hospital, Gil Hospital, Eulji General Hospital, Chonnam National University Hospital, and Busan Paik Hospital. The patients/participants provided their written informed consent to participate in this study.

## Author contributions

SS designed and helped in the data analysis and manuscript writing. SS, TO, and HC contributed to the conception of the study. SS and CK performed the data analyses and wrote the manuscript. EB, K-HO, SH, and SM collected the data. SM and SK helped perform the analysis with constructive discussions. All authors contributed to the article and approved the submitted version.

## Funding

This work was supported by the Research Program funded by the Korea Centers for Disease Control and Prevention (2011E3300300, 2012E3301100, 2013E3301600, 2013E3301601, 2013E3301602, 2016E3300200, 2016E3300201, 2016E3300202, 2019E320100, 2019E320101, 2019E320102, and 2022-11-007), by the National Research Foundation of Korea (NRF) funded by the Korea government (MSIT) (NRF-2020R1F1A1074001 and NRF-2019R1A2C2086276), and a grant (BCRI21042 and BCRI22079) of Chonnam National University Hospital Biomedical Research Institute.

## Conflict of interest

The authors declare that the research was conducted in the absence of any commercial or financial relationships that could be construed as a potential conflict of interest.

## Publisher's note

All claims expressed in this article are solely those of the authors and do not necessarily represent those of their affiliated organizations, or those of the publisher, the editors and the reviewers. Any product that may be evaluated in this article, or claim that may be made by its manufacturer, is not guaranteed or endorsed by the publisher.
