# Supplementary material for: Serum triglycerides level is independently associated with renal outcomes in patients with non-dialysis chronic kidney disease: Results from KNOW-CKD study
Source: Front Nutr. 2022 Nov 24;9:1037618. doi: 10.3389/fnut.2022.1037618 (PMC9729769; doi:10.3389/fnut.2022.1037618)
Supplement: Supplementary file 1 [file Data_Sheet_1.docx]

**- Supplementary Materials -**

**Serum triglycerides level is independently associated with renal outcomes in patients with non-dialysis chronic kidney disease: results from KNOW-CKD study**

Sang Heon Suh^1^, Tae Ryom Oh^1^, Hong Sang Choi^1^, Chang Seong Kim^1^, Eun Hui Bae^1^, Kook-Hwan Oh^2^, Seung Hyeok Han^3^, Seong Kwon Ma^1,^*, and Soo Wan Kim^1,^*, on behalf of the Korean Cohort Study for Outcomes in Patients With Chronic Kidney Disease (KNOW-CKD) Investigators

^1^Department of Internal Medicine, Chonnam National University Medical School and Chonnam National University Hospital, Gwangju, Korea

^2^Department of Internal Medicine, Seoul National University Hospital, Seoul, Korea

^3^Department of Internal Medicine, College of Medicine, Institute of Kidney Disease Research, Yonsei University, Seoul, Korea

**Running title:** TG and renal outcomes in CKD

**Corresponding authors**

*Seong Kwon Ma, M.D., Ph.D., Department of Internal Medicine, Chonnam National University Medical School, 42 Jebongro, Gwangju 61469, Korea, Tel: +82-62-220-6579, Fax: +82-62-225-8578, Email: drmsk@hanmail.net

*Soo Wan Kim, M.D., Ph.D., Department of Internal Medicine, Chonnam National University Medical School, 42 Jebongro, Gwangju 61469, Korea, Tel: +82-62-225-6271, Fax: +82-62-220-8578, Email: skimw@chonnam.ac.kr

**Table of contents**

Supplementary Table S1. Cox regression analysis of serum TG levels for secondary outcomes

Supplementary Table S2. Cox regression analysis of serum TG levels for primary outcome in the subjects excluding CKD stage 1

Supplementary Table S3. Cox regression analysis of serum TG levels for primary outcome in the subjects excluding CKD stage 5

Supplementary Table S4. Cox regression analysis of serum TG-to-HDL-C ratio for primary outcome

Supplementary Table S5. Cause-specific hazard model for secondary outcomes by serum TG levels

Supplementary Table S6. Cox regression analysis of serum TG levels for decline of kidney function in various subgroups

Supplementary Table S7. Cox regression analysis of serum TG levels for onset of ESRD in various subgroups

Supplementary Figure S1. Comparison of he rates of renal function decline per year by serum TG levels

Supplementary Figure S2. Kaplan-Meier analysis for cumulative incidence of decline of kidney function by serum TG levels

Supplementary Figure S3. Kaplan-Meier analysis for cumulative incidence of onset of ESRD by serum TG levels

Supplementary Figure S4. Restricted cubic spline of serum TG level on decline of kidney function

Supplementary Figure S5. Restricted cubic spline of serum TG level on onset of ESRD

Supplementary Figure S6. Cumulative incidence (Gray's test for competing risk) of composite renal event by serum TG levels

**Supplementary Table S1. Cox regression analysis of serum TG levels for secondary outcomes**

|  | Serum TG level | Events, n (%) | Model 1 | | Model 2 | | Model 3 | | Model 4 | |
| --- | --- | --- | --- | --- | --- | --- | --- | --- | --- | --- |
|  |  |  | HR  (95%CIs) | *P* value | HR  (95%CIs) | *P* value | HR  (95%CIs) | *P* value | HR  (95%CIs) | *P* value |
| Decline of kidney function | Q1 | 114 (21.3) | Reference |  | Reference |  | Reference |  | Reference |  |
|  | Q2 | 133 (24.9) | 1.239  (0.964, 1.591) | 0.093 | 1.209  (0.927, 1.575) | 0.161 | 1.357  (1.016, 1.813) | 0.039 | 1.222  (0.910, 1.640) | 0.183 |
|  | Q3 | 148 (27.6) | 1.457  (1.141, 1.860) | 0.003 | 1.296  (0.992, 1.693) | 0.058 | 1.581  (1.168, 2.141) | 0.003 | 1.506  (1.109, 2.044) | 0.009 |
|  | Q4 | 171 (31.7) | 1.736  (1.369, 2.202) | < 0.001 | 1.622  (1.240, 2.122) | < 0.001 | 2.121  (1.465, 3.070) | < 0.001 | 1.699  (1.167, 2.474) | 0.006 |
| Onset of ESRD | Q1 | 119 (22.3) | Reference |  | Reference |  | Reference |  | Reference |  |
|  | Q2 | 150 (28.0) | 1.327  (1.043, 1.688) | 0.013 | 1.372  (1.064, 1.767) | 0.015 | 1.457  (1.102, 1.925) | 0.008 | 1.293  (0.970, 1.725) | 0.080 |
|  | Q3 | 173 (32.3) | 1.607  (1.272, 2.030) | < 0.001 | 1.480  (1.146, 1.912) | 0.003 | 1.533  (1.141, 2.059) | 0.005 | 1.103  (0.819, 1.487) | 0.518 |
|  | Q4 | 176 (32.7) | 1.640  (1.299, 2.070) | < 0.001 | 1.407  (1.081, 1.832) | 0.011 | 1.786  (1.246, 2.560) | 0.002 | 1.272  (0.891, 1.815) | 0.186 |

Note: Model 1, unadjusted model. Model 2, model 1 + adjusted for age, sex, Charlson comorbidity index, primary renal disease, smoking status, medications (ACEi/ARBs, diuretics, number of antihypertensive drugs, statins), BMI, WC, SBP and DBP. Model 3, model 2 + adjusted for hemoglobin, albumin, fasting glucose, total cholesterol, LDL-C, HDL-C, 25(OH) vitamin D, hs-CRP, and CACS Model 4, model 3 + CKD stage and spot urine ACR. Abbreviations: CI, confidence interval; HR, hazard ratio; TG, triglycerides; Q1, 1^st^ quartile; Q2, 2^nd^ quartile; Q3, 3^rd^ quartile; Q4, 4^th^ quartile.

**Supplementary Table S2. Cox regression analysis of serum TG levels for primary outcome in the subjects excluding CKD stage 1**

|  | Serum TG level | Events, n (%) | Model 1 | | Model 2 | | Model 3 | | Model 4 | |
| --- | --- | --- | --- | --- | --- | --- | --- | --- | --- | --- |
|  |  |  | HR  (95%CIs) | *P* value | HR  (95%CIs) | *P* value | HR  (95%CIs) | *P* value | HR  (95%CIs) | *P* value |
| Composite renal event | Q1 | 160 (38.6) | Reference |  | Reference |  | Reference |  | Reference |  |
|  | Q2 | 187 (41.6) | 1.113  (0.901, 1.374) | 0.321 | 1.209  (0.969, 1.508) | 0.093 | 1.305  (1.023, 1.665) | 0.032 | 1.231  (0.959, 1.582) | 0.103 |
|  | Q3 | 212 (45.5) | 1.302  (1.060, 1.598) | 0.012 | 1.261  (1.009, 1.575) | 0.041 | 1.414  (1.093, 1.829) | 0.008 | 1.135  (0.873, 1.475) | 0.346 |
|  | Q4 | 226 (47.9) | 1.398  (1.141, 1.712) | 0.001 | 1.299  (1.034, 1.632) | 0.025 | 1.757  (1.277, 2.419) | < 0.001 | 1.443  (1.048, 1.986) | 0.024 |

Note: Model 1, unadjusted model. Model 2, model 1 + adjusted for age, sex, Charlson comorbidity index, primary renal disease, smoking status, medications (ACEi/ARBs, diuretics, number of antihypertensive drugs, statins), BMI, WC, SBP and DBP. Model 3, model 2 + adjusted for hemoglobin, albumin, fasting glucose, total cholesterol, LDL-C, HDL-C, 25(OH) vitamin D, hs-CRP, and CACS Model 4, model 3 + CKD stage and spot urine ACR. Abbreviations: CI, confidence interval; HR, hazard ratio; TG, triglycerides; Q1, 1^st^ quartile; Q2, 2^nd^ quartile; Q3, 3^rd^ quartile; Q4, 4^th^ quartile.

**Supplementary Table S3. Cox regression analysis of serum TG levels for primary outcome in the subjects excluding CKD stage 5**

|  | Serum TG level | Events, n (%) | Model 1 | | Model 2 | | Model 3 | | Model 4 | |
| --- | --- | --- | --- | --- | --- | --- | --- | --- | --- | --- |
|  |  |  | HR  (95%CIs) | *P* value | HR  (95%CIs) | *P* value | HR  (95%CIs) | *P* value | HR  (95%CIs) | *P* value |
| Composite renal event | Q1 | 142 (28.3) | Reference |  | Reference |  | Reference |  | Reference |  |
|  | Q2 | 161 (32.3) | 1.212  (0.937, 1.519) | 0.095 | 1.254  (0.989, 1.590) | 0.061 | 1.361  (1.049, 1.766) | 0.020 | 1.184  (0.906, 1.548) | 0.217 |
|  | Q3 | 182 (36.8) | 1.492  (1.198, 1.858) | < 0.001 | 1.330  (1.048, 1.689) | 0.019 | 1.499  (1.135, 1.979) | 0.004 | 1.390  (1.054, 1.834) | 0.020 |
|  | Q4 | 211 (41.1) | 1.741  (1.407, 2.155) | < 0.001 | 1.483  (1.165, 1.889) | 0.001 | 1.881  (1.335, 2.651) | < 0.001 | 1.517  (1.075, 2.141) | 0.018 |

Note: Model 1, unadjusted model. Model 2, model 1 + adjusted for age, sex, Charlson comorbidity index, primary renal disease, smoking status, medications (ACEi/ARBs, diuretics, number of antihypertensive drugs, statins), BMI, WC, SBP and DBP. Model 3, model 2 + adjusted for hemoglobin, albumin, fasting glucose, total cholesterol, LDL-C, HDL-C, 25(OH) vitamin D, hs-CRP, and CACS Model 4, model 3 + CKD stage and spot urine ACR. Abbreviations: CI, confidence interval; HR, hazard ratio; TG, triglycerides; Q1, 1^st^ quartile; Q2, 2^nd^ quartile; Q3, 3^rd^ quartile; Q4, 4^th^ quartile.

**Supplementary Table S4. Cox regression analysis of serum TG-to-HDL-C ratio for primary outcome**

|  | Serum TG/HDL-C ratio | Events, n (%) | Model 1 | | Model 2 | | Model 3 | | Model 4 | |
| --- | --- | --- | --- | --- | --- | --- | --- | --- | --- | --- |
|  |  |  | HR  (95%CIs) | *P* value | HR  (95%CIs) | *P* value | HR  (95%CIs) | *P* value | HR  (95%CIs) | *P* value |
| Composite renal event | Q1 | 163 (30.8) | Reference |  | Reference |  | Reference |  | Reference |  |
|  | Q2 | 191 (35.9) | 1.286  (1.019, 1.623) | 0.034 | 1.228  (0.963, 1.566) | 0.097 | 1.249  (0.957, 1.631) | 0.101 | 1.134  (0.864, 1.489) | 0.365 |
|  | Q3 | 224 (42.1) | 1.846  (1.475, 2.311) | < 0.001 | 1.501  (1.170, 1.926) | 0.001 | 1.730  (1.265, 2.366) | 0.001 | 1.204  (0.875, 1.656) | 0.254 |
|  | Q4 | 228 (42.7) | 1.759  (1.397, 2.215) | < 0.001 | 1.550  (1.194, 2.013) | 0.001 | 1.935  (1.314, 2.850) | 0.001 | 1.623  (1.106, 2.383) | 0.013 |

Note: Model 1, unadjusted model. Model 2, model 1 + adjusted for age, sex, Charlson comorbidity index, primary renal disease, smoking status, medications (ACEi/ARBs, diuretics, number of antihypertensive drugs, statins), BMI, WC, SBP and DBP. Model 3, model 2 + adjusted for hemoglobin, albumin, fasting glucose, total cholesterol, LDL-C, HDL-C, 25(OH) vitamin D, hs-CRP, and CACS Model 4, model 3 + CKD stage and spot urine ACR. Abbreviations: CI, confidence interval; HDL-C, high density lipoprotein cholesterol; HR, hazard ratio; TG, triglycerides; Q1, 1^st^ quartile; Q2, 2^nd^ quartile; Q3, 3^rd^ quartile; Q4, 4^th^ quartile.

**Supplementary Table S5. Cause-specific hazard model for secondary outcomes by serum TG levels**

|  | Serum TG level | Model 1 | | Model 2 | | Model 3 | | Model 4 | |
| --- | --- | --- | --- | --- | --- | --- | --- | --- | --- |
|  |  | HR  (95%CIs) | *P* value | HR  (95%CIs) | *P* value | HR  (95%CIs) | *P* value | HR  (95%CIs) | *P* value |
| Decline of kidney function | Q1 | Reference |  | Reference |  | Reference |  | Reference |  |
|  | Q2 | 1.239  (0.965, 1.590) | 0.093 | 1.209  (0.926, 1.578) | 0.163 | 1.357  (1.007, 1.829) | 0.045 | 1.222  (0.899, 1.661) | 0.201 |
|  | Q3 | 1.457  (1.142, 1.859) | 0.002 | 1.296  (0.987, 1.700) | 0.061 | 1.581  (1.148, 2.177) | 0.005 | 1.506  (1.103, 2.057) | 0.009 |
|  | Q4 | 1.737  (1.370, 2.201) | < 0.001 | 1.622  (1.236, 2.129) | < 0.001 | 2.122  (1.426, 3.156) | < 0.001 | 1.699  (1.129, 2.558) | 0.011 |
| Onset of ESRD | Q1 | Reference |  | Reference |  | Reference |  | Reference |  |
|  | Q2 | 1.327  (1.040, 1.692) | 0.022 | 1.372  (1.058, 1.778) | 0.017 | 1.457  (1.092, 1.944) | 0.010 | 1.294  (0.964, 1.737) | 0.087 |
|  | Q3 | 1.607  (1.268, 2.035) | < 0.001 | 1.480  (1.135, 1.931) | 0.004 | 1.533  (1.125, 2.089) | 0.007 | 1.103  (0.810, 1.503) | 0.533 |
|  | Q4 | 1.640  (1.296, 2.075) | < 0.001 | 1.408  (1.061, 1.868) | 0.018 | 1.787  (1.240, 2.574) | 0.002 | 1.273  (0.876, 1.850) | 0.206 |

Note: Model 1, unadjusted model. Model 2, model 1 + adjusted for age, sex, Charlson comorbidity index, primary renal disease, smoking status, medications (ACEi/ARBs, diuretics, number of anti-HTN drugs, statins), BMI, WC, SBP and DBP. Model 3, model 2 + adjusted for hemoglobin, albumin, fasting glucose, total cholesterol, LDL-C, HDL-C, 25(OH) vitamin D, hs-CRP, and CACS. Model 4, model 3 + CKD stage and spot urine ACR. Abbreviations: CI, confidence interval; ESRD, end-stage renal disease; HR, hazard ratio; TG, triglycerides; Q1, 1^st^ quartile; Q2, 2^nd^ quartile; Q3, 3^rd^ quartile; Q4, 4^th^ quartile.

**Supplementary Table S6. Cox regression analysis of serum TG levels for decline of kidney function in various subgroups**

|  | Serum TG levels | Events, n (%) | Unadjusted HR  (95%CIs) | *P* for interaction | Adjusted HR  (95%CIs) | *P* for interaction |
| --- | --- | --- | --- | --- | --- | --- |
| **Age < 60 years** | Q1 | 82 (23.0) | Reference | 0.235 | Reference | 0.046 |
|  | Q2 | 86 (24.9) | 1.155 (0.853, 1.563) |  | 1.068 (0.743, 1.534) |  |
|  | Q3 | 91 (27.7) | 1.398 (1.037, 1.884) |  | 1.2117 (0.831, 1.784) |  |
|  | Q4 | 123 (35.8) | 1.884 (1.424, 2.493) |  | 1.610 (1.019, 2.543) |  |
| **Age ≥ 60 years** | Q1 | 32 (18.1) | Reference |  | Reference |  |
|  | Q2 | 47 (24.9) | 1.440 (0.919, 2.257) |  | 1.820 (1.042, 3.179) |  |
|  | Q3 | 57 (27.4) | 1.604 (1.039, 2.475) |  | 2.309 (1.318, 4.044) |  |
|  | Q4 | 48 (24.6) | 1.471 (0.939, 2.303) |  | 1.904 (0.927, 3.910) |  |
| **Male** | Q1 | 64 (21.8) | Reference | 0.235 | Reference | 0.640 |
|  | Q2 | 70 (22.7) | 1.071 (0.763, 1.503) |  | 1.007 (0.743, 1.365) |  |
|  | Q3 | 87 (24.9) | 1.301 (0.942, 1.797) |  | 1.240 (0.944, 1.629) |  |
|  | Q4 | 111 (30.2) | 1.591 (1.169, 2.165) |  | 1.514 (1.172, 1.955) |  |
| **Female** | Q1 | 50 (20.8) | Reference |  | Reference |  |
|  | Q2 | 63 (27.9) | 1.457 (1.005, 2.113) |  | 1.334 (0.864, 2.060) |  |
|  | Q3 | 61 (32.6) | 1.694 (1.165, 2.462) |  | 1.622 (1.018, 2.584) |  |
|  | Q4 | 60 (35.1) | 1.988 (1.365, 2.894) |  | 1.760 (0.987, 3.135) |  |
| **BMI < 23 kg/m^2^** | Q1 | 63 (25.3) | Reference | 0.899 | Reference | 0.239 |
|  | Q2 | 50 (25.4) | 1.168 (0.805, 1.693) |  | 1.084 (0.663, 1.773) |  |
|  | Q3 | 37 (28.9) | 1.470 (0.978, 2.209) |  | 1.200 (0.667, 2.160) |  |
|  | Q4 | 27 (29.3) | 1.609 (1.023, 2.530) |  | 2.585 (1.195, 5.594) |  |
| **BMI ≥ 23 kg/m^2^** | Q1 | 51 (18.1) | Reference |  | Reference |  |
|  | Q2 | 83 (24.9) | 1.355 (0.956, 1.921) |  | 1.470 (0.981, 2.204) |  |
|  | Q3 | 109 (27.0) | 1.549 (1.111, 2.160) |  | 1.819 (1.225, 2.700) |  |
|  | Q4 | 144 (32.4) | 1.931 (1.403, 2.658) |  | 1.762 (1.110, 2.795) |  |
| **eGFR ≥ 45 mL/min./1.73m^2^** | Q1 | 45 (14.2) | Reference | 0.236 | Reference | 0.022 |
|  | Q2 | 42 (15.1) | 1.140 (0.749, 1.736) |  | 1.123 (0.677, 1.863) |  |
|  | Q3 | 33 (12.9) | 1.035 (0.660, 1.623) |  | 0.915 (0.515, 1.627) |  |
|  | Q4 | 56 (22.1) | 1.829 (1.235, 2.710) |  | 2.045 (1.016, 4.119) |  |
| **eGFR < 45 mL/min./1.73m^2^** | Q1 | 69 (31.9) | Reference |  | Reference |  |
|  | Q2 | 91 (35.5) | 1.103 (0.806, 1.509) |  | 1.322 (0.901, 1.939) |  |
|  | Q3 | 115 (41.1) | 1.349 (1.001, 1.819) |  | 1.787 (1.220, 2.619) |  |
|  | Q4 | 115 (40.2) | 1.377 (1.201, 1.857) |  | 1.708 (1.070, 2.724) |  |
| **Spot urine ACR < 300 mg/g** | Q1 | 53 (17.5) | Reference | 0.054 | Reference | 0.043 |
|  | Q2 | 46 (18.2) | 1.015 (0.684, 1.507) |  | 0.788 (0.478, 1.300) |  |
|  | Q3 | 37 (16.7) | 1.008 (0.662, 1.535) |  | 0.990 (0.563, 1.741) |  |
|  | Q4 | 24 (13.0) | 0.851 (0.525, 1.380) |  | 1.100 (0.481, 2.517) |  |
| **Spot urine ACR ≥ 300 mg/g** | Q1 | 59 (27.4) | Reference |  | Reference |  |
|  | Q2 | 84 (32.2) | 1.330 (0.953, 1.855) |  | 1.361 (0.914, 2.026) |  |
|  | Q3 | 110 (37.5) | 1.577 (1.149, 2.165) |  | 1.620 (1.092, 2.404) |  |
|  | Q4 | 141 (42.0) | 1.717 (1.267, 2.328) |  | 1.897 (1.199, 3.002) |  |

Note: The model was adjusted for age, sex, Charlson comorbidity index, primary renal disease, smoking status, medications (ACEi/ARBs, diuretics, number of antihypertensive drugs, statins), BMI, WC, SBP, DBP, hemoglobin, albumin, fasting glucose, total cholesterol, LDL-C, HDL-C, 25(OH) vitamin D, hs-CRP, CACS, CKD stage, and spot urine ACR. Abbreviations: ACR, albumin-to-creatinine ratio; BMI, body mass index; CI, confidence interval; Cr, creatinine; eGFR, estimated glomerular filtration rate; HR, hazard ratio; TG, triglycerides.

**Supplementary Table S7. Cox regression analysis of serum TG levels for onset of ESRD in various subgroups**

|  | Serum TG levels | Events, n (%) | Unadjusted HR  (95%CIs) | *P* for interaction | Adjusted HR  (95%CIs) | *P* for interaction |
| --- | --- | --- | --- | --- | --- | --- |
| **Age < 60 years** | Q1 | 68 (19.0) | Reference | 0.034 | Reference | 0.096 |
|  | Q2 | 93 (26.9) | 1.507 (1.102, 2.061) |  | 1.494 (1.019, 2.190) |  |
|  | Q3 | 102 (31.1) | 1.862 (1.369, 2.531) |  | 1.077 (0.710, 1.634) |  |
|  | Q4 | 120 (34.9) | 2.128 (1.580, 2.868) |  | 1.663 (1.036, 2.670) |  |
| **Age ≥ 60 years** | Q1 | 51 (28.8) | Reference |  | Reference |  |
|  | Q2 | 57 (30.2) | 1.059 (0.726, 1.545) |  | 1.222 (0.738, 2.023) |  |
|  | Q3 | 71 (34.1) | 1.215 (0.848, 1.742) |  | 1.200 (0.731, 1.970) |  |
|  | Q4 | 56 (28.7) | 1.014 (0.694, 1.483) |  | 1.109 (0.576, 2.133) |  |
| **Male** | Q1 | 72 (24.5) | Reference | 0.236 | Reference | 0.282 |
|  | Q2 | 83 (26.9) | 1.128 (0.823, 1.547) |  | 0.898 (0.681, 1.184) |  |
|  | Q3 | 106 (30.4) | 1.371 (1.016, 1.849) |  | 0.952 (0.738, 1.228) |  |
|  | Q4 | 114 (31.0) | 1.346 (1.002, 1.809) |  | 1.009 (0.783, 1.301) |  |
| **Female** | Q1 | 47 (19.6) | Reference |  | Reference |  |
|  | Q2 | 67 (29.6) | 1.640 (1.129, 2.383) |  | 1.897 (1.193, 3.017) |  |
|  | Q3 | 67 (35.8) | 2.004 (1.378, 2.914) |  | 1.387 (0.849, 2.266) |  |
|  | Q4 | 62 (36.3) | 2.250 (1.537, 3.294) |  | 1.650 (0.900, 3.024) |  |
| **BMI < 23 kg/m^2^** | Q1 | 53 (21.3) | Reference | 0.134 | Reference | 0.464 |
|  | Q2 | 61 (31.0) | 1.706 (1.179, 2.467) |  | 1.545 (0.952, 2.509) |  |
|  | Q3 | 50 (39.1) | 2.344 (1.590, 3.456) |  | 1.752 (0.978, 3.139) |  |
|  | Q4 | 32 (34.8) | 2.164 (1.393, 3.363) |  | 1.491 (0.702, 3.168) |  |
| **BMI ≥ 23 kg/m^2^** | Q1 | 65 (23.1) | Reference |  | Reference |  |
|  | Q2 | 89 (26.7) | 1.112 (0.808, 1.531) |  | 1.150 (0.779, 1.697) |  |
|  | Q3 | 122 (30.2) | 1.331 (0.985, 1.799) |  | 1.039 (0.721, 1.498) |  |
|  | Q4 | 144 (32.4) | 1.444 (1.077, 1.936) |  | 1.491 (0.702, 3.168) |  |
| **eGFR ≥ 45 mL/min./1.73m^2^** | Q1 | 13 (4.1) | Reference | 0.013 | Reference | 0.054 |
|  | Q2 | 23 (8.2) | 2.168 (1.098, 4.280) |  | 3.092 (1.326, 7.207) |  |
|  | Q3 | 15 (5.9) | 1.644 (0.782, 3.456) |  | 1.508 (0.554, 4.104) |  |
|  | Q4 | 29 (11.5) | 3.214 (1.669, 6.191) |  | 3.740 (1.237, 11.307) |  |
| **eGFR < 45 mL/min./1.73m^2^** | Q1 | 106 (49.1) | Reference |  | Reference |  |
|  | Q2 | 127 (49.6) | 0.982 (0.758, 1.271) |  | 1.192 (0.869, 1.634) |  |
|  | Q3 | 158 (56.4) | 1.142 (0.892, 1.462) |  | 1.056 (0.768, 1.452) |  |
|  | Q4 | 147 (51.4) | 1.081 (0.841, 1.389) |  | 1.070 (0.729, 1.571) |  |
| **Spot urine ACR < 300 mg/g** | Q1 | 33 (10.9) | Reference | 0.241 | Reference | 0.140 |
|  | Q2 | 46 (18.2) | 1.684 (1.076, 2.636) |  | 1.525 (1.029, 2.260) |  |
|  | Q3 | 32 (14.4) | 1.437 (0.883, 2.339) |  | 1.462 (0.959, 2.229) |  |
|  | Q4 | 20 (10.8) | 1.156 (0.662, 2.020) |  | 1.900 (1.109, 3.256) |  |
| **Spot urine ACR ≥ 300 mg/g** | Q1 | 84 (39.1) | Reference |  | Reference |  |
|  | Q2 | 100 (38.3) | 1.039 (0.777, 1.388) |  | 1.147 (0.809, 1.626) |  |
|  | Q3 | 136 (46.4) | 1.245 (0.948, 1.634) |  | 0.862 (0.611, 1.216) |  |
|  | Q4 | 150 (44.6) | 1.144 (0.876, 1.494) |  | 1.086 (0.728, 1.618) |  |

Note: The model was adjusted for age, sex, Charlson comorbidity index, primary renal disease, smoking status, medications (ACEi/ARBs, diuretics, number of antihypertensive drugs, statins), BMI, WC, SBP, DBP, hemoglobin, albumin, fasting glucose, total cholesterol, LDL-C, HDL-C, 25(OH) vitamin D, hs-CRP, CACS, CKD stage, and spot urine ACR. Abbreviations: ACR, albumin-to-creatinine ratio; BMI, body mass index; CI, confidence interval; Cr, creatinine; eGFR, estimated glomerular filtration rate; HR, hazard ratio; TG, triglycerides.


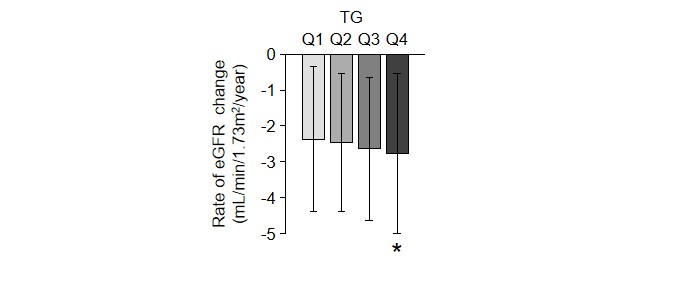


**Supplementary Figure S1. Comparison of he rates of renal function decline per year by serum TG levels**

Note: Error bars indicate standard deviation. *, *P* < 0.05 *versus* Q1 by one-way ANOVA with Scheffe’s *post-hoc* analyses. Abbreviations: eGFR, estimated glomerular filtration rate; TG, triglycerides; Q1, 1^st^ quartile; Q2, 2^nd^ quartile; Q3, 3^rd^ quartile; Q4, 4^th^ quartile.

**
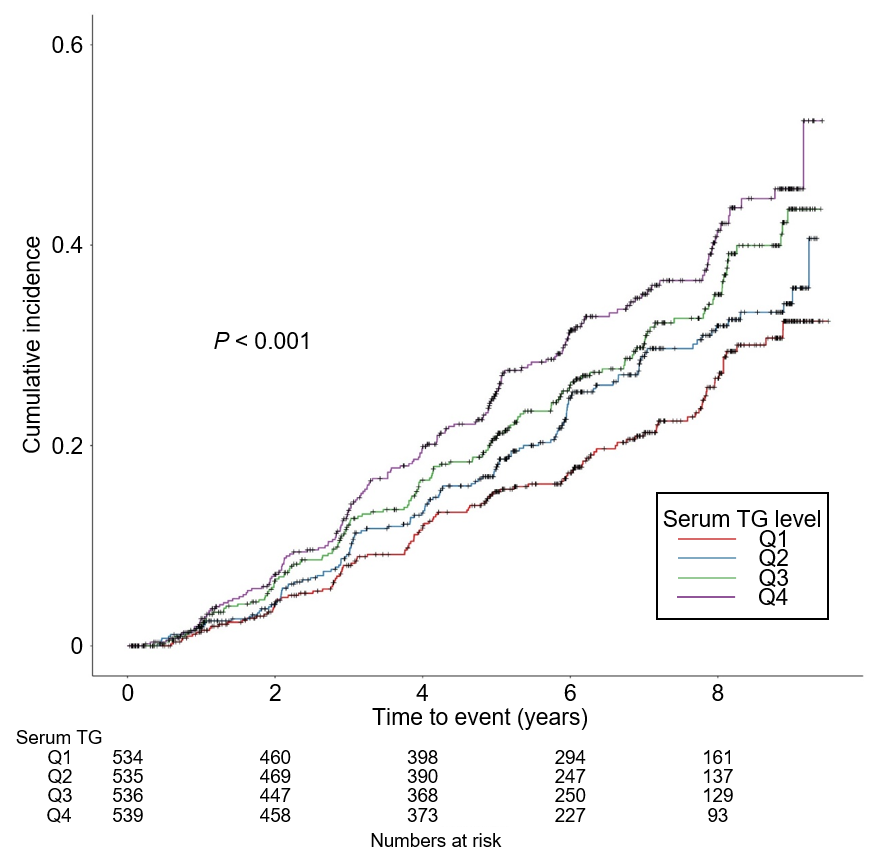
**

**Supplementary Figure S2. Kaplan-Meier analysis for cumulative incidence of decline of kidney function by serum TG levels**

Note: *P* value by Log-rank test. Abbreviations: TG, triglycerides; Q1, 1^st^ quartile; Q2, 2^nd^ quartile; Q3, 3^rd^ quartile; Q4, 4^th^ quartile.

**
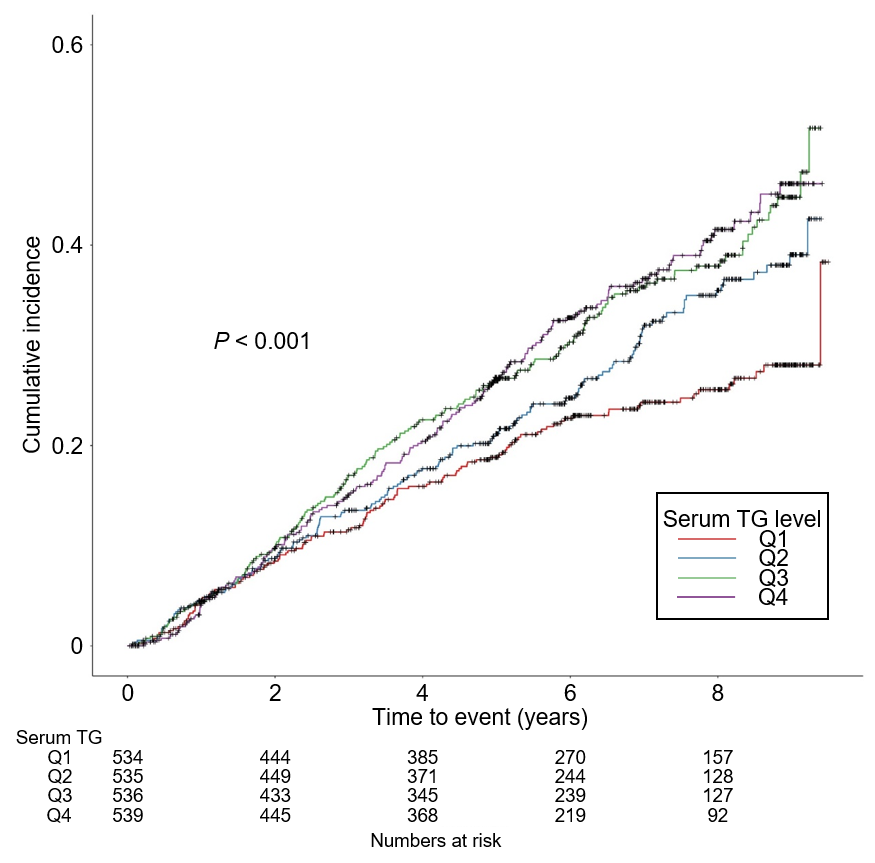
**

**Supplementary Figure S3. Kaplan-Meier analysis for cumulative incidence of onset of ESRD by serum TG levels**

Note: *P* value by Log-rank test. Abbreviations: TG, triglycerides; Q1, 1^st^ quartile; Q2, 2^nd^ quartile; Q3, 3^rd^ quartile; Q4, 4^th^ quartile.

**
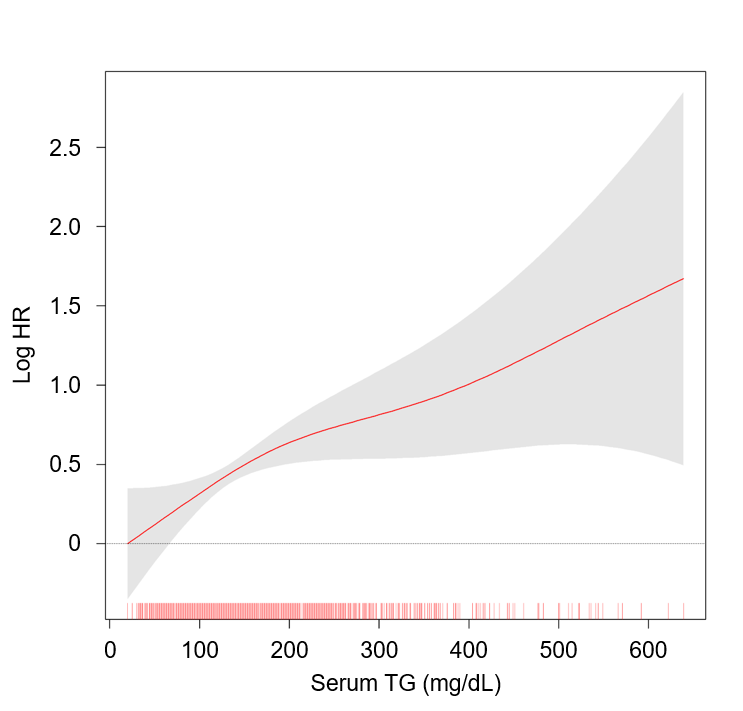
**

**Supplementary Figure S4. Restricted cubic spline of serum TG level on decline of kidney function**

Note: Adjusted HR of serum TG level as a continuous variable for decline of kidney function is depicted. The model was adjusted for age, sex, Charlson comorbidity index, primary renal disease, smoking status, medications (ACEi/ARBs, diuretics, number of antihypertensive drugs, statins), BMI, WC, SBP, DBP, hemoglobin, albumin, fasting glucose, total cholesterol, LDL-C, HDL-C, 25(OH) vitamin D, hs-CRP, CACS, CKD stage, and spot urine ACR. Abbreviations: HR, hazard ratio. TG, triglycerides.

**
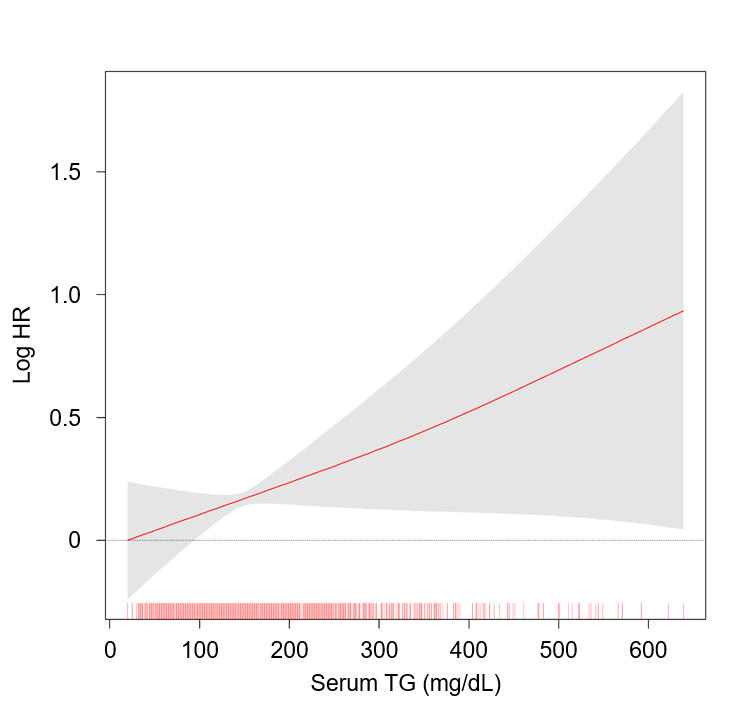
**

**Supplementary Figure S5. Restricted cubic spline of serum TG level on onset of ESRD**

Note: Adjusted HR of serum TG level as a continuous variable for onset of ESRD is depicted. The model was adjusted for age, sex, Charlson comorbidity index, primary renal disease, smoking status, medications (ACEi/ARBs, diuretics, number of antihypertensive drugs, statins), BMI, WC, SBP, DBP, hemoglobin, albumin, fasting glucose, total cholesterol, LDL-C, HDL-C, 25(OH) vitamin D, hs-CRP, CACS, CKD stage, and spot urine ACR. Abbreviations: HR, hazard ratio. TG, triglycerides.

**
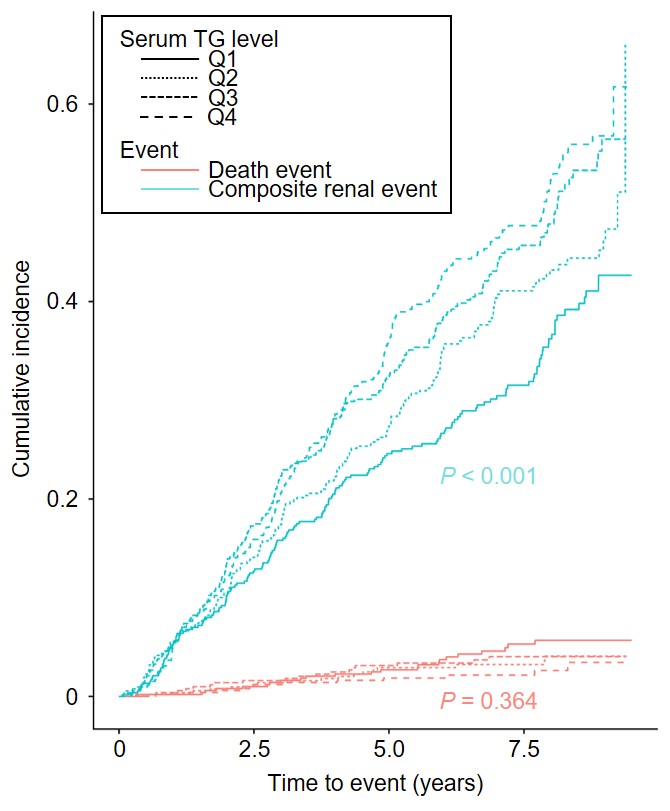
**

**Supplementary Figure S6. Cumulative incidence (Gray's test for competing risk) of composite renal event by serum TG levels**

Note: *P* value by Gray's test. Abbreviations: TG, triglycerides; Q1, 1^st^ quartile; Q2, 2^nd^ quartile; Q3, 3^rd^ quartile; Q4, 4^th^ quartile.
